# Can Metabolomic Approaches Become a Tool for Improving Early Plant Disease Detection and Diagnosis with Modern Remote Sensing Methods? A Review

**DOI:** 10.3390/s23125366

**Published:** 2023-06-06

**Authors:** Anton Terentev, Viktor Dolzhenko

**Affiliations:** All-Russian Institute of Plant Protection, 196608 Saint Petersburg, Russia

**Keywords:** metabolomics, Raman spectroscopy, hyperspectral remote sensing, GC-MS, LC-MS, early plant disease detection, phytoalexins, plant metabolome

## Abstract

The various areas of ultra-sensitive remote sensing research equipment development have provided new ways for assessing crop states. However, even the most promising areas of research, such as hyperspectral remote sensing or Raman spectrometry, have not yet led to stable results. In this review, the main methods for early plant disease detection are discussed. The best proven existing techniques for data acquisition are described. It is discussed how they can be applied to new areas of knowledge. The role of metabolomic approaches in the application of modern methods for early plant disease detection and diagnosis is reviewed. A further direction for experimental methodological development is indicated. The ways to increase the efficiency of modern early plant disease detection remote sensing methods through metabolomic data usage are shown. This article provides an overview of modern sensors and technologies for assessing the biochemical state of crops as well as the ways to apply them in synergy with existing data acquisition and analysis technologies for early plant disease detection.

## 1. Introduction

In the context of climate change, the rapid globalization of the world’s economy and the world’s population growth, the limitation of crop production worldwide, which is caused by biotic factors such as pests and diseases, has become a significant economic and social risk factor [1,2]. For example, average worldwide yield losses caused by pests and diseases in grain crops such as wheat, rice and maize are estimated to be 21.5%, 30.0%, and 22.6%, respectively [3]. The actual losses from plant diseases only, caused by fungi, oomycetes, bacteria, and viruses have been estimated to account for 16% of the attainable crop production worldwide [4].

The economic losses in agriculture and horticulture from various pests and pathogens keep growing and amount to billions of US dollars worldwide. FAO estimates that between 20 and 40 percent of global crop production is lost to pests and plant diseases and it costs the global economy around USD 300 billion annually [5]. For example, the losses associated with citrus greening, caused by the bacterium *Candidatus liberibacter*, are estimated to be over USD 1 billion per year only in Florida, USA [6]. At the same time, the generally accepted methods for detecting and diagnosing plant diseases, such as visual estimation for detecting and diagnosing plant diseases; microscopic diagnosing of pests and pathogens through morphological features; and molecular, serological, and microbiological diagnostic methods do not meet the modern requirements put forward by precision agriculture [7,8,9,10].

In the last two decades, new remote sensing methods and approaches for plant disease control have been created. They are based on various types of new devices that allow the timely detection of plant pests and diseases to then take necessary control measures [11,12]. Digital technologies such as big data systems, mathematical and statistical analysis, artificial intelligence, and machine learning also play an important role in solving pest control problems [13].

The main areas using modern technical means in agriculture are agriculture for ecosystem services, phenotyping, agricultural land use monitoring, crop yield forecasting, and crop monitoring for yield optimization (precision farming) [8,14,15]. The main tasks for modern technical means in precision farming include weed [16,17,18] and disease [8,19] detection and diagnosis, nutrient [20,21] and water stress [22,23] detection, and soil property diagnosis for their optimization [24,25]. Among these tasks, the accurate and early estimation of plant pest and disease spreading and harmfulness is one of the most important for intensive crop production, the breeding of new varieties, and pesticide usage regulation [8].

In recent years, the number of articles on the early detection of plant diseases using modern approaches such as hyperspectral remote sensing, GC-MS and HPLC-MS chromatography, and Raman spectroscopy has grown significantly. However, the number of applied systems for early plant disease diagnosis based on such data is still negligible. The need to properly extract the critical data to identify diseases from the collected dataset is the main gap preventing the creation of such systems [26]. However, within the relevant disciplines, there are no methodologies for developing criteria to select such data [8,13,26]. Thus, to create systems for early plant disease detection using modern technical methods, an interdisciplinary approach is required.

Metabolomics is a powerful tool for studying various aspects of plant physiology and biology, which significantly expands our knowledge of the metabolic and molecular regulatory mechanisms that regulate plant growth, development, and response to stress, as well as improving the yield and the quality of crops [27]. We believe that the analysis of metabolomic data makes it possible to identify groups of metabolites, whose concentration significantly changes during the development of a disease. It should be noted that these changes may already begin on the first day after inoculation [28]. Determining such specific compounds allows refinement of the data obtained by technical methods.

In this paper, we wanted to analyze the advantages and disadvantages of modern remote sensing technologies for the creation of stable early plant disease detection systems and how to overcome them. Another scientific assumption we tried to verify was that new remote sensing technical methods have insufficient accuracy in diagnosing plant diseases due to a lack of comparison of the remote sensing data obtained and the biochemical processes occurring as a result of plant–pathogen interactions. Thus, the main objective of our paper was to show the perspectives of applying metabolomics approaches to identify biomarkers to create systems for early plant disease detection on the basis of remote sensing data.

Within this analysis, the available results are summarized and the main gaps in the field of the early detection of plant diseases with modern technical methods are highlighted.

## 2. Molecular Methods for Early Plant Disease Detection in Plant Protection

In plant protection, the PCR and qPCR methods are used to identify pathogens that cause plant diseases. Whole genome sequencing has become an effective method to investigate the information contained in the genome sequence of plant pathogens. On the basis of DNA sequence data analysis, species and genus specific primers and probes have been developed to assess the qualitative and quantitative content of pathogens in studied plants tissues [29,30]. The PCR method is based on the repeated doubling of a certain section of DNA with the help of enzymes under artificial conditions (in vitro). As a result, DNA amounts sufficient for visual detection are produced. Only the area that satisfies the specified conditions is copied, and only if it is present in the sample studied. In applied plant protection, PCR is typically used to determine the species of a pathogen in a known diseased sample [30,31].

Real-time PCR (qPCR, RT-qPCR) is a proven tool that uses genomics and transcriptomics approaches to create tests used for the laboratory detection of plant pathogens by analyzing DNA obtained from test samples. Using qPCR, it is possible to determine not only the presence of RNA or DNA in a sample, but also its amount in real time. The method has high sensitivity—the proportion of correctly identified diseases by the test— and high specificity—the proportion of correctly identified samples without the disease [32,33].

The qPCR method is similar to conventional PCR; however, fluorescent labels or an intercalating dye are added to the reaction. The amount of DNA can be estimated from the intensity of luminescence or staining. For this, curves of dependence of signal intensity on time are constructed. There are two ways to detect PCR products: non-specific, using dyes, and specific, using DNA probes [34,35].

The main advantages of qPCR are high sensitivity and specificity, a higher test speed compared to conventional PCR, a wide range of detectable infections, and the ability to quantify pathogens and make tests for mixed infections due to the ability to detect several pathogens in one test [36,37,38].

The disadvantages of using qPCR in plant protection include the need for the long-term processing of samples by qualified personnel. The in-field molecular diagnosis of plant pathogens is still unavailable, and the need for simultaneous DNA and RNA tests for some crops further complicates this task. Attempts to adapt the technologies applicable to the analysis of human pathogens have not yet become widespread in plant protection for various reasons [39,40,41,42]. When handling specimens, one must be qualified to determine which diseases may potentially be present and have a set of necessary probes to perform the tests. When diagnosing plant diseases at an early stage in the absence of visible symptoms, the problem of detecting diseased plants has to be solved by a random choice [39,41]. The qPCR method is still too expensive for the routine diagnosis of plant diseases, and using probes that include a wide variety of pathogens would make the tests even more expensive. The sample preparation method is destructive, which, in some cases, is also a disadvantage [9].

## 3. Metabolomics, GC-MS/MS, and LC-MS/MS Chromatography

Metabolomics is the scientific study of chemical processes involving metabolites, low-molecular-weight substrates, intermediates, and products of cellular metabolism [43,44]. Plants produce a large number of metabolites, which play an important role in their growth, development, and environmental response. These metabolites are divided into primary and secondary, or specific metabolites [27,45,46]. Primary metabolites such as sugars, amino acids, and organic acids are essential for plant growth and development. Specific metabolites such as alkaloids, phenols, polyphenols, terpenes, and polyamides are critical for environmental interactions [28]. Primary metabolites are very similar in structure and prevalence, while specific metabolites vary widely across the plant kingdom. However, the influence of biotic and abiotic factors changes the quantitative content of both types of metabolites [47,48]. The most common methods for obtaining metabolomic data are chromatography and spectroscopy. Currently, GC-MS/MS and LC-MS/MS are the most used techniques for metabolome acquisition [49].

Chromatography is a method for separating and analyzing mixtures of substances, as well as studying the physicochemical properties of substances. It is based on the principle of sorption. The analyte is distributed between two phases: mobile (liquid or gaseous eluent) and immobile (liquid or solid sorbent). Various components of the mixture interact differently with adsorbents, allowing accurate conclusions about the quantitative and qualitative composition of the mixture to be drawn. According to the physical nature of the mobile and stationary phases, liquid and gas chromatography are distinguished. The usage of chromatography for plant metabolome study allows the solving of a number of problems, such as phenotyping, the determination of abiotic and biotic stresses in plants, the control of pesticides, etc. [49,50,51].

In plant protection, different types of chromatography are used to study different groups of substances. None of the existing methods can compare and evaluate the content of all the cellular metabolites; therefore, as a rule, a combination of different methods is used [28,52,53]. In modern research on early plant disease detection through metabolome analysis, chromatography is used to obtain the primary data. The disease detection is carried out both through specific biomarkers, which are the product of a specific metabolome of target plant, and through changes in the quantitative and percentage content of primary metabolites. Pontes et al. (2016) used a biomarker combination of NMR and chemometrics for citrus huanglongbing (HLB) identification [54]. Galeano Garcia et al. (2018) discovered that the LC-MS metabolite profiles reliably discriminated between early and late asymptomatic infection [55]. Dai et al. (2019) used PLS-DA, OPLS, and ANOVA, analyzing untargeted GC-MS-obtained metabolomics data for the early diagnosis of strawberry anthracnose caused by *Colletotrichum theobromicola* [56]. Canas et al. (2020) developed and validated an HPLC-based method for the quantification of gallic acid, ferulic acid, epicatechin, taxifolin, rutin, resveratrol, and secoisolariciresinol in pine tissues for pine wilt disease detection [57]. Medic et al. (2021) studied the phenolic response to walnut anthracnose (*Ophiognomonia leptostyla*) using UHPLC MS/MS. A total of 26 phenolic compounds were identified and quantified, mostly flavanols, flavonols, and naphthoquinones [58]. Di Masi et al. (2022) used HPLC-ESI-Q-TOF-MS to detect differences between healthy and infected olive trees. Different metabolites, such as flavonoids and long-chain fatty acids were identified as potential specific biomarkers for “olive quick decline syndrome” [59]. Deshaies et al. (2022) used UHPLC-QTOF-MS on wheat spikes to provide information on how chitosan might provide protection or stimulate wheat resistance to infection by *F. graminearum* [60]. The listed works show the great potential of mass spectrometry techniques for studying different features of plant–pathogen interactions.

The main advantage of chromatography is the ability to analyze samples with unknown composition mixtures, i.e., the simultaneous separation and analysis of substances. Good uniformity and reproducibility are achieved by high sample separation efficiency, since there are multiple processes of sorption–desorption. Metabolomic data complements the data of genomic and transcriptomic studies greatly [49,61,62].

The main disadvantages of chromatography are the high requirements for sample preparation and the cost of equipment and consumables. To obtain data on all the substance classes contained in the sample, the usage of different types of chromatography is required. Processing the data of metabolomic studies requires complex multivariate mathematical analysis. Another significant drawback for this area is the current lack of comprehensive metabolite library databases [49,61,62]. However, the complex quantitative metabolomic analysis of plants and plant pathogen metabolites has not yet become widespread. Therefore, metabolomic methods in study of plant pathology lag behind genomic and transcriptomic methods [52].

We also wanted to mention such a method of metabolome analysis as the use of electronic nose devices, designed to capture volatile organic compounds (VOCs). The e-nose device contains a large number of gas sensors that can detect a significant range of VOCs. Compared with traditional GC-MS or LC-MS techniques, electronic noses are noninvasive and can be a rapid, cost-effective tool for early plant disease detection [49,63]. The researchers using this approach typically combine the e-nose data with chromatography data [64,65] and use machine learning techniques to process the results [65,66]. Although more than 15 years have passed since the first articles on this topic appeared, this area remains poorly studied. There is also no reliable data that volatile compounds can serve as biomarkers for the majority of most harmful plant diseases. Some authors believe that it would be a challenge to identify such diseases based on VOC emission only [67,68]. However, in the area of phytophage–plant–entomophage interactions, volatile compounds such as terpenes, salicylates, phenylpropanoids, and other VOCs undoubtedly play a leading role [68,69,70,71,72]. We believe that further research in this area can be of great benefit in applying electronic nose technology to improve existing biological plant protection systems in industrial greenhouses.

## 4. New Technical Methods in Plant Protection

A variety of advanced technical methods are currently used in the agricultural industry to receive and process data to detect plant pathogens. The current review will consider the most promising methods for obtaining such data, selected from an analysis of the available literature [8,9,73]. The most promising methods for data acquisition are spectroscopy and optical imaging. As for results processing and analysis, various data processing methods are used.

The main criteria for choosing the methods described below were the ability of the method to quickly acquire and process the data necessary for early plant disease diagnostics.

### 4.1. Optical Remote Sensing

Optical remote sensing methods used in agriculture include RGB imaging, multi- and hyperspectral imaging, thermography, and fluorescence imaging. Recently, the performance and availability of these types of sensors have increased significantly. In addition, a significant number of articles has been published on their usage [73,74]. An important feature of optical sensors is their ability to quickly acquire data from large areas. This is achieved through aircraft and satellite usage. Thus, these sensors may become a solution to the problem of plant disease detection on large areas of agricultural land [9]. As for the early diagnosis of plant diseases, not all types of optical sensors are optimal for this task [8,9,74].

Thermography makes it possible to study the changes in temperature of the studied object and thus to track the qualitative changes occurring in it. Thermographic sensors are commonly thermal imaging cameras which create 2D images capturing infrared (IR) radiation [50,75]. Some authors considered this method for early plant disease diagnosis. Oerke et al. (2006) studied the detection of cucumber downy mildew caused by the oomycete *Pseudoperonospora cubensis*. The study discovered that infrared thermography could serve as a suitable tool for disease analysis under controlled conditions. However, outdoors, this method did not provide an acceptable accuracy due to the variability of leaf temperature modified by environmental conditions [76]. Stoll et al. (2008), on the contrary, managed to obtain high accuracy when detecting the fungal pathogen *Plasmopara viticola* on grapevine [77]. Another study by Oerke et al. (2011), on apple scab caused by the fungi *Venturia inaequalis*, showed that thermographic measurements can reveal differences in disease severity resulting from disease stage, resistance of host tissue, and differences in the aggressiveness of *V. inaequalis* isolates [78].

Regardless, thermography allows the examination of large areas quickly using thermal imaging cameras and is a good tool for detecting plant diseases, but it is not suitable for early diagnosis due to the fact that the symptoms of different types of biotic and abiotic stress look very similar on thermographic images. In addition, this method is negatively affected by changes in ambient temperature. For the same reasons, the use of this technology to detect diseases at early stages is hardly possible, especially in the case of latent or concomitant infections [9,51,79].

Fluorescence imaging allows the study of changes in the photosynthetic activity of plants and thus detect the presence of pathogens. Devices for fluorescence imaging are usually active sensors with an LED or laser light source. The most common method to measure chlorophyll fluorescence uses pulse amplitude modulation (PAM) fluorometry [80,81]. This method was described in the following articles. Rodrıguez-Moreno et al. (2008) was able to perform the early detection of bean infection by the bacterium *Pseudomonas syringae* using red chlorophyll fluorescence that was measured using the kinetic imaging chlorophyll fluorometer FluorCam (Photon Systems Instruments, Brno, Czech Republic) [82]. Baurigel et al. (2014) studied wheat head blight caused by the *Fusarium* spp. fungi. The authors obtained good results both in laboratory and field measurements. Under laboratory conditions, chlorophyll fluorescence imaging was able to early detect even very low levels of infection (ca. 5%) as early as the sixth day after inoculation, while visual classification was only possible beginning from seventh day after inoculation [83].

Although fluorescence imaging cannot cover large areas due to technical limitations, in laboratory studies, this method allows the detection of plant diseases at early stages before visible symptoms appear. However, plants under biotic and abiotic stresses may look very similar when using this technique. In addition, following a strict sample preparation protocol is needed for fluorometry. Thus, the difficulties and the disadvantages of the method make it nearly impossible for common early plant disease detection in agriculture [9,80].

Sun-induced chlorophyll fluorescence is a promising new direction in the remote sensing of plants. The Earth Explorer-Fluorescence Explorer (FLEX) mission, a European Space Agency (ESA) mission, can map vegetation fluorescence to quantify photosynthetic activity which will lead to better insights into crop health and stress [84,85]. Interesting new data have already been obtained in such areas of remote sensing as nitrogen uptake [86], fundamental vegetation trait quantification [87], and drought stress [88].

The advantages and disadvantages of this remote sensing technique are discussed in reviews [89,90], while studies [91,92] show the possibility of its practical application. However, authors believe that current data to assess the potential of this technique for the early detection of plant diseases are insufficient.

RGB imaging uses the RGB range to acquire 2D images of a selected object in order to study its changes. The significant increase in the resolution of RGB cameras, along with the increase in their availability, has made it possible to obtain HD images [40,93]. Commercial satellites of the latest generations can also obtain very-high-resolution imagery [94]. Obtaining data of such a high quality allows us to confidently recognize the visual manifestations of plant diseases, both by the standard method of human expert rating or using various automation tools. However, despite all the advantages of the RGB imaging method, it is difficult to use it for early plant disease detection, due to the fact that many diseases do not have any visual symptoms at an early stage [95,96]. In addition, there is an issue of very similar changes in leaf color and texture induced by abiotic and biotic stresses, which makes their accurate diagnosis nearly impossible [97,98].

Multi- and hyperspectral imaging are the most promising among the optical imaging techniques for early plant disease detection and diagnosis. Multispectral sensors collect data from a small number (usually 3–15) of spectral ranges. Hyperspectral sensors use hundreds of channels within which high-resolution information is collected and recorded independently. These bands cover a wide range of wavelengths ranging from 400 to 2500 nm: the VIS range (400–700 nm), the NIR range (700–1100 nm), and the SWIR range (1100–2500) [26,99,100]. In recent years, a large number of different hyperspectral sensors covering these ranges has become available for scientifical and practical use, including satellite-based ones [101,102].

Hyperspectral imaging offers many more opportunities for early plant disease detection because it provides image data with very high spectral resolution that can help with the accurate and timely determination of the physiological status of agricultural crops [103]. In recent years, the number of studies on early plant disease detection using hyperspectral imaging has increased significantly [104]. These studies prove that hyperspectral imaging can detect diseases caused by fungal [105,106,107], viral [108,109,110], and bacterial pathogens [111,112,113] as well as various abiotic stresses [114,115]. The data on the most studied crops (citrus fruits, nightshades, oil palm, and wheat) were reviewed in detail by Terentev et al. (2022). The authors of the review mentioned that despite the presence of a large number of articles on early plant disease detection using hyperspectral remote sensing, no unified methods for detecting diseases in respective specific wavelength ranges have been developed yet [26].

Summing up, we can highlight the following advantages of optical sensors. Firstly, this is the ability to quickly obtain data from large areas (with the exception of fluorescence imaging), including data from satellites and aircrafts [9]. Secondly, the low cost, high availability, and prevalence of sensors (only for RGB imaging) [40]. Thirdly, the presence of many specific vegetation indices, which make it possible to quickly and easily solve various agronomic problems, including the determination of the phytosanitary state of plants [116].

The main disadvantage of optical imaging is the difficulty to accurately diagnose diseases, including latent and mixed infections [9]. In addition, the disadvantages of optical sensors include the high requirements for the automation of large data volume analysis [26,117]. In the case of satellites, clouds are a common problem, which can make it impossible to obtain data at the time needed [118]. Multispectral and hyperspectral sensors may be too expensive for small farm usage [119].

We believe that the most promising and relevant areas of optical imaging are RGB and hyperspectral imaging. RGB imaging can already solve many problems with detecting diseases and plant pests. There are specialized software products that make it easier for agronomists to identify plant diseases using RGB imaging. These are applications for identifying greenhouse pests such as the Syngenta Pest Management App or apps for crop protection specialists such as Agrio or AgroAI [120]. The use of high-quality satellite photos makes it possible to monitor weeds, pests, and disease outbreaks over vast areas. However, due to very similar changes in leaf color induced by plant pathogens and the absence of visible symptoms in some cases, RGB imaging cannot act as a tool for early plant disease diagnosis [9,10].

Hyperspectral remote sensing is one of the most promising tools for diagnosing plant diseases at an early stage [8,10]. Hyperspectral snapshot cameras have the potential to create systems for detecting and diagnosing plant disease in large agricultural areas. However, there are existing gaps that prevent the creation of such systems [9,26].

The low resolution on current hyperspectral cameras is one of the factors hindering successes in their application [119]. This complicates the task of using them for the early detection of diseases when receiving data from satellites and UAVs. In addition, at the moment, there are no hyperspectral snapshot cameras operating in the VIS-NIR-SWIR ranges simultaneously, and at least two different devices are required to capture data from large areas (for example, Cubert S185 for the 400–1000 nm range and Specim SWIR for the 1000–2500 nm range). Existing hyperspectral sensors which operate in all the three ranges simultaneously, such as the ASD FieldSpec 4 spectroradiometer, which operates in the wavelength range of 350–2500 nm, are push-broom cameras, with all the ensuing shortcomings [26]. This factor is technical and it is likely to be overcome in the future, the same as the current high cost of hyperspectral sensors.

As for the gaps in fundamental knowledge, we believe that the area of leaf–light interactions is the most important topic, which is not studied enough [121]. An additional difficulty hindering the solution of this problem is that different plant pathogens can develop in different zones of leaf tissues. The existing models of leaf–light interactions are highly simplified for the tasks put forward by the direction of the optical imaging of plant–pathogen interactions and cannot definitely identify diagnostic errors that occur during direct and internal light reflection and absorption analysis [121].

### 4.2. Spectroscopy

Spectroscopy is a branch of science that studies the spectra of electromagnetic radiation as a function of wavelength or frequency, measured by spectrographic equipment and other methods, to obtain information about the structure and properties of the studied matter. There is a wide variety of spectroscopy techniques that are used in various fields of study. UV-VIS-NIR spectroscopy, infrared spectroscopy (IR), fluorescence spectroscopy (FS), and Raman spectroscopy (RS) are the most used in plant protection studies [9,73,74].

UV-VIS-NIR spectroscopy is a method used to determine the optical properties (transmittance, reflectance, and absorbance) of liquids and solids. It operates in the optical range between 175 nm and 3300 nm. The technique measures the absorption of light across the desired optical range [74,122].

In publications devoted to the detection of plant diseases, instead of UV/VIS/NIR spectroscopy, authors sometimes use the terms VIS/NIR or NIRS spectroscopy, depending on the equipment used and its optical range. In recent years, multiple works that reveal the possibility of successfully detecting plant diseases at an early stage with this method have appeared [110,123,124,125,126].

In their study, Morellos et al. (2020) were aiming to develop an algorithm for tomato chlorosis virus (ToCV) detection using VIS-NIR spectrometry. The authors mentioned that ELISA and RT-PCR were the current conventional methods for ToCV detection. The authors managed to reach up to 85% early classification accuracy of ToCV when applying ANN to VIS-NIR spectroscopy data [110]. Nijar and Abu-Khalaf (2021) were able to reach up to 100% early classification accuracy of tomato gray mold caused by the anamorph fungus *Botrytis cinerea*. The results of VIS-NIR spectroscopy were verified using PCR. The authors used PCA as the data analysis tool [123]. Lelong et al. (2010) used VIS-NIR spectroscopy to evaluate the severity of oil palm basal stem rot caused by the fungus *Ganoderma boninense*. A classification accuracy of 94% was achieved using PLS-DA [124]. Hou et al. (2022) studied tomato late blight caused by the oomycete *Phytophthora infestans*. The study combined VIS-NIR spectroscopy with machine learning. The classification accuracy reached up to 99% [125]. Tu et al. (2022) managed to determine early drought stress of tomato with VIS-NIR spectroscopy data. The authors used 1D-SP-Net as the main classification tool, which outperformed 1D-CNN, partial least squares discriminant analysis (PLSDA), and random forest (RF) models, demonstrating an accuracy of 96.3% [126].

The advantage of UV-VIS-NIR spectroscopy is that it is suitable for the determination of a wide analyte concentration variety in a solution. In addition, the quantification of analytes in solutions using UV/VIS/NIR is simpler and less time-consuming than chromatographic analysis [74,122]. The disadvantage of this method is that chromatographic analysis is more accurate and precise than UV/VIS/NIR. Another very important disadvantage is that some components in a sample solution may interfere with other components, which makes the research results questionable [9,122].

Fluorescence spectroscopy is a type of electromagnetic spectroscopy that analyzes the fluorescence of a sample. It uses a beam of light, usually ultraviolet (wavelength from 10 to 400 nm), that excites the electrons in the molecules of certain compounds and causes them to emit light. The devices that measure fluorescence are called fluorometers [50].

FS can be used for early plant disease detection, which has been shown in a number of studies [127,128,129,130]. Belasque et al. (2007) and Lins et al. (2008) studied citrus canker caused by the bacteria *Xanthomonas axonopodis pv. citri* with laser fluorescence spectroscopy (LIF) and managed to detect diseased leaves at early stages [127,128]. In their study, Sankaran et al. (2012) were able to detect citrus greening caused by the bacterium *Candidatus Liberibacter asiaticus* at early stages of the disease. Naïve-Bayes and bagged decision tree classifiers reached more than 85% and 94% detection accuracy, respectively [129]. Sallem et al. (2020) managed to detect citrus canker on grapefruits using LIF. Principal component analysis (PCA) and partial least square regression (PLSR) both showed excellent results at classifying the disease at early stages [130]. The main advantage of the FS method is that it can detect the concentration of a component with a sensitivity around 1000 times greater than that of most spectrophotometric methods. The major challenge for FS is photobleaching [9]. Photobleaching is a general term for any photochemical process that causes the molecule to be permanently unable to fluoresce. This phenomenon results in decreased sensitivity, and inaccurate recording and data collection, and its influence was observed in the application of FS in early plant disease detection. Although there are ways to circumvent this limitation, FS, despite its advantages, has not yet become widely used in the field of plant protection [50,131].

IR spectroscopy refers to vibrational spectroscopy. It utilizes the concept that molecules tend to absorb specific frequencies of light that are characteristic of the corresponding structure of the molecules. IR radiation is absorbed by the molecules at specific frequencies depending on the molecular bonds between atoms and the types of atoms at the ends of the bonds. Analysis of the infrared spectrum of absorption or emission allows a determination of the chemical composition of the sample [132].

Fourier-transform infrared (FTIR) spectrometers are the most common instruments used for IR spectroscopy. FTIR measures the absorbance of infrared light of a sample and generates a spectrum based on the functional groups in the material. The difference between IR and FTIR is that IR is constructed from a raw signal and FTIR is constructed from an interferogram. IR takes a single spectrum, whereas FTIR employs an interferometer and takes a number of scans. IR uses monochromatic light and FTIR uses polychromatic light [133].

In the last decade, only a small number of works have been published on early plant disease detection using IR and FTIR spectrometry [134,135,136,137]. In their study, Sankaran et al. (2010) used an InfraSpec VFA-IR spectrometer (Wilks Enterprise Inc., East Norwalk, CT, USA) to collect the mid-infrared spectra in the range of 5.15–10.72 m (1942–933 cm^−1^) with a 0.04 m resolution. The authors used quadratic discriminant analysis (QDA) and k-nearest neighbors (kNN) as a classifier tool to detect citrus greening caused by the bacteria *Candidatus liberibacter* spp. The performance of the kNN-based algorithm (higher than 95%) was better than the QDA-based algorithm [134]. Salman et al. (2010) and Erukhimovitch et al. (2010) used an FTIR spectrometer (Bruker Tensor 127) to study if it is possible to detect four different soil fungi genera: *Rhizoctonia*, *Colletotrichum*, *Verticillium*, and *Fusarium oxysporum*, which may cause serious damage to a large number of crops. The authors used pure fungi cultures for FTIR spectroscopy measurements. The measurements were performed using FTIR–ATR with a liquid-nitrogen-cooled mercury-cadmium-telluride MCT detector in the wave region 600–4000 cm^−1^, with a spectral resolution of 4 cm^−1^ [135,136]. Hawkins et al. (2010) made a comparison of citrus greening with other citrus diseases using the FTIR technique. ATR spectra were collected using a Thermoelectron Nicolet (Madison, WI) Magna 850 FTIR spectrometer with a deuterated triglycine sulfate (DTGS) detector. The spectra were acquired at 2 cm^−1^ resolutions [137].

One of the major gaps of IR-based spectroscopy techniques is the need for complicated sample preparation. The removal of water is a typical step in the sample preparation for IR spectroscopy because water is highly IR active. This complicates the work, increases the personnel requirements, and thus practically eliminates the method’s advantages [9,73,138]. For this reason, although handheld FTIR spectrometers already exist, their application in agronomy does not include the field of plant protection, but is limited to the analysis of soil conditions [139].

The advantage of this area of spectroscopy is that IR and FTIR spectrometers are non-destructive and highly sensitive. They are capable of identifying organic functional groups and often specific organic compounds. IR spectroscopy can be quantitative with appropriate standards and uniform sample thicknesses. There are handheld FTIR spectrometers that can be used for field diagnostics. IR spectroscopy is complementary to Raman spectroscopy [9,133,140].

The downsides of IR spectroscopy are limited surface sensitivity and the requirement for standard usage for sample quantitation. The identification of mixtures/multiple sample components may require additional laboratory preparations and analyses. The biggest disadvantage of the method is that water strongly absorbs infrared light which may interfere with the analysis of dissolved, suspended, or wet samples. This makes it extremely difficult to obtain data from the cytoplasm and extracellular fluid of plant tissues, and thus makes it almost impossible to use handheld IR and FTIR devices in the field [9,132,139].

Raman spectroscopy is based on inelastic photon scattering, known as Raman scattering. Laser light interacts with the vibrations of atoms in molecules, phonons, or other excitations in the system, as a result of which the energy of laser photons is shifted to the region of high or low values. This energy shift provides information about the vibrational modes in the system. Infrared spectroscopy usually provides similar but additional information [132,141]. The main difference between Raman and IR spectroscopy is that Raman spectroscopy depends on a change in polarizability of a molecule, whereas IR spectroscopy depends on a change in the dipole moment. Raman spectroscopy measures the relative frequencies at which a sample scatters radiation, whereas IR spectroscopy measures absolute frequencies at which a sample absorbs radiation [133,141].

In general, most of the molecules that have symmetry manifest themselves in both infrared and Raman spectra. Molecules with an inversion center are a special case. If the molecule has an inversion center, then Raman and IR will be mutually exclusive, that is, the bond will be active in either Raman or IR spectra. There is a general rule that functional groups with strong changes in the dipole moment are clearly visible in IR spectra, while functional groups with weak changes or with a high degree of symmetry are more visible in Raman spectra [132].

Raman spectroscopy has a number of advantages over IR and FTIR for plant disease studies. It can more easily investigate carbon bonds in aliphatic and aromatic rings. In addition, RS can be used to identify molecules with bonds that are difficult to see in IR spectra (for example, O–O, S–H, C=S, N=N, C=C, etc.). Raman spectroscopy is more suitable for studying reactions in aqueous media (water has a very small Raman cross-section, allowing for spectral acquisition from cytoplasm and extracellular fluid) [9,140].

In most RS-based plant disease studies, the authors used hand-held Raman spectrometers, as this is the most suitable for a future practical application. A number of authors have proved that Raman spectroscopy usage can determine plant diseases caused by all types of pathogens, e.g., viral [142,143,144,145], bacterial [146,147,148,149], and fungal [150,151,152]. The main aspects of Raman spectroscopy usage for plant disease detection were discussed in detail in the review by Farber et al. (2019). However, a unified system for detecting plant diseases via this method has not yet been developed [9].

The advantage of Raman spectroscopy is that it is non-destructive and highly sensitive. There are handheld Raman spectrometers that can be used for in-field diagnostics. Raman spectrometers are capable of identifying organic functional groups and specific organic compounds. Raman spectroscopy can be quantitative with appropriate standards and uniform sample thicknesses. IR spectroscopy is complementary to Raman spectroscopy, which is important for the development of the method.

The main disadvantage of Raman spectroscopy is that it can hardly be used to study highly fluorescent samples. The identification of sample components in some cases may require laboratory preparations and analyses and cannot be performed with a handheld device. Although there are some libraries for compound and mixture identifications, the information on plant metabolites is not yet exhaustive.

### 4.3. Digital Technologies

New technical methods used in plant sciences generate a huge amount of data about the studied objects, both plants and pathogens. Therefore, automatic means of data processing and analysis are used for their adaptation to the needs of agriculture [73], wherein different methods and approaches are suitable for different purposes. When creating primers and probes for PCR, bioinformatics approaches are used [153]. When analyzing spectrometry, chromatography, and optical sensor data, diseased plant health data may not be analyzed correctly using parametric approaches such as simple or multiple regression and functional analysis; therefore, non-parametric approaches are used [154]. The commonest types of non-parametric classifiers used for diseased and healthy plant determination are principal component analysis (PCA), support vector machine (SVM), cluster analysis (CA), partial least-square (PLS), and artificial neural network (ANN) [155,156]. When processing chromatographic and spectrometric data, in addition to non-parametric classifiers, databases are also used to determine recognizable substances and compounds [157,158,159]. The choice of an analysis algorithm depends on many factors, such as data amount, the presence of a visible feature’s ability to be distinguished, and so on [160]. Therefore, the correct approach to the choice of instruments for classification is one of the most important success factors in early plant disease remote sensing.

When analyzing data from optical sensors, spectral vegetation indices (SVI) are often used. The SVI obtained from remote sensing are simple and effective tools for quantitative and qualitative evaluations of vegetation cover, vigor, and growth dynamics. Specific SVI can be used to detect certain plant diseases based on formulae including disease-specific wavebands [114,161].

The big data obtained through spectrometry, chromatography, and optical sensors contain everything necessary for early plant disease detection. At the same time, the methods of analyzing this data such as machine learning, neural networks, and statistical and manual analysis, despite their huge applied role, are only automation methods and do not make a significant contribution to solving the problem of early plant disease detection [26,162].

## 5. Discussion

The first purpose of this article was to review the most promising approaches and technical methods for early plant disease detection and diagnosis. We believe that among current technical methods, hyperspectral remote sensing and Raman spectrometry are most suitable for these purposes. A comparison of these two methods with the proven qPCR method is shown in Table 1.

The main advantage of hyperspectral remote sensing is the ability to collect high-precision data from large areas [8]. This makes this method a leader in disease monitoring, leaving the possibility of developing diagnostic systems. The main advantages of Raman spectrometry are quickness, non-destructiveness, the absence of sample preparation, and the possibility of using handheld devices in field conditions [9]. A potential advantage of hyperspectral sensing is its high sensitivity and specificity [163]. An additional potential advantage of Raman spectrometry is that a telescope addition can increase the spectra collection range to over 60 m, which may allow large area monitoring [164,165].

The main disadvantage of both methods is the much lower current sensitivity and specificity than those of PCR [8,9,166]. Unlike PCR, these methods are not generally accepted and approved, and there are no documented systems for plant disease detection on this basis.

Thus, the main task in the development of both methods should be a sensitivity and specificity increase to create plant disease recognition systems that are not inferior to the PCR method. At the same time, in order to achieve the goals of precise agriculture, it is highly desirable that such systems would not have the disadvantages of PCR, namely the requirements for personnel skills, destructiveness, and long sample preparation [8,9,73,163].

The second purpose of this article was to identify current gaps in scientific knowledge that hinder the creation of early plant disease detection systems using new technical methods and to spotlight the ways to overcome them.

In our previous review on hyperspectral remote sensing, we concluded that one cannot rely on technical analysis only to properly select the important wavelengths needed for disease identification [26]. We believe that this statement also applies to Raman spectrometry. The problem both in hyperspectral remote sensing and Raman spectrometry data lies in the current lack of methodology to determine the data that reliably characterize a disease [26]. We believe that studies of plant and plant pathogen metabolomics can help solve this problem by discovering biomarkers, which could be a “fingerprint” of a particular disease.

We suppose that the selection of specific metabolites, which are supposed to be used as biomarkers for determining plant diseases, is fundamental [166,167]. We believe that comparative analysis of metabolomes is important to identify groups of plant metabolites whose concentration changes significantly during disease development [46,47,48,49]. Depending on the chemical nature of these compounds, either liquid or gas chromatography can be used for their study.

Plant disease development is associated with changes in primary and specialized metabolites from the first hours after inoculation [28]. At the same time, changes in the concentration of specific metabolites can be associated not only with plant pathogen development, but also with other stress factors. In this case, some of the changes may be nonspecific [167,168]. The identification of key components that significantly change during disease development is usually carried out using principal component analysis, PCA [45,169].

As a result of metabolome analysis, the most representative group of compounds to assess a disease’s development is revealed from the obtained metabolite variety. It is possible to develop approaches for early plant disease detection based on the identified physicochemical compound properties. Unfortunately, there are a number of limitations. Firstly, such metabolites as, for example, sugars, amino acids, and flavonoids, may change under any adverse impact. The data obtained from those changes can only be used for the evaluation of a plant’s physiological state as a whole [28]. Secondly, special metabolites differ in each crop; hence, it is necessary to choose a method for their rapid analysis each time [28,167,168]. Thirdly, PCA, and other technical analysis methods, have a number of disadvantages when used in biology [170,171]. Replacing these methods with a more thorough analysis will require a lot more time and human resources. On the other hand, it should result in a better compound group description as the characteristics of a disease.

In the articles on hyperspectral remote sensing, the authors usually do not compare the obtained wavelengths with the presence of certain metabolites in the studied diseased plants [26,163]. The study of Gold et al. (2020) is a rare example of hyperspectral remote sensing usage to analyze the changes of certain groups of metabolites in the studied plant [107]. In this study, the authors analyzed how groups of compounds such as carbohydrates (lignin, sugars, and cellulose), protein, and chlorophyll correspond to certain wavebands in the VIS-NIR spectrum during the development of late and early blight on potato. However, these compounds are primary metabolites that on their own may not be enough to serve as characteristic markers of a particular pathogen for early plant disease detection [172,173,174]. A recent study by Terentev et al. (2023) showed that the main changes in the wheat metabolome upon inoculation with *Puccinia triticina* are manifested in UV and SWIR ranges and cannot be managed by a VIS-NIR hyperspectral camera [160]. In addition, UV hyperspectral remote sensing data were recently successfully compared with metabolomics data in a study by Brugger et al. (2023) [175].

As was mentioned in the section on hyperspectral remote sensing, specific vegetation indices (SVI) are also used to determine certain aspects of the phytosanitary state of plants, including the content of certain groups of substances. Most of the existing SVI make it possible to measure such parameters as chlorophyll, pigment and water content, biomass density, and some biophysical parameters of plants [116]. However, although the number of SVI using the biophysical parameters of plants is quite large, their percentage measuring the content of secondary metabolites is critically small. For this reason, such indices are usually used as an auxiliary tool to assess the condition of plants [82]. Moreover, most articles on hyperspectral remote sensing generally bypass the question of obtained important band comparison with any compounds. This can be explained by the fact that there are almost no databases and publications on the accordance of spectra with specific metabolites or compound groups.

In the articles on Raman spectrometry, the authors, on the contrary, usually indicate several compounds and groups of compounds as specific biomarkers of the studied diseases. A summary of the substances mentioned in these studies is shown in Table 2.

As follows from Table 2, these compounds belong to both primary and special metabolites. Some of them may be involved in both roles, depending on the host plant. However, it should be noted that similar changes in primary and secondary plant metabolites can occur as a result of various influences, including various types of biotic and abiotic stresses. This includes changes in the content of carbohydrates, proteins, carotenoids, lignins, cellulose, aliphatics, phenolics, pectin, xylans, and chlorophyll [46,47,48]. The changes in the content of ketones, phenolics, terpens, and flavonoids may act as biomarkers for specific pathogens.

As a response to the diseases, different plant families produce different specific metabolites that are specific to certain types of pathogens. For example, in potatoes, these are phytoalexins such as terpenes, rishitin and lubimin, and ketone solavetivone [179,180]. In wheat, these are phenylamide compounds [181,182]. We believe that such specific compounds, together with the overall picture of metabolomic changes, should become biomarkers for the early detection of plant diseases. Some of the recent studies already include a comparison of chromatography and Raman spectrometry data, but so far do not take into account the features of specific metabolites responsible for the interaction with pathogens [183].

Despite the potential importance of metabolomics, molecular methods usage is also very important for accurately measuring both the qualitative and quantitative composition of a pathogen in a plant [184,185], and the verification of the pathogen diagnosis results [186]. It should be noted that in most studies, except for fungal infections, PCR or qPCR are used to verify Raman spectrometry data [142,143,144,145,146,147,148,149,150,151]. In contrast to RS, in hyperspectral remote sensing studies, the molecular methods of disease confirmation are statistically used rarely, being limited to other disease severity determination methods [26].

Therefore, to create systems for early plant disease detection, it is necessary to use modern technical methods as well as metabolomics approaches and PCR verification. The combination of data from various technical methods could significantly speed up the search for biomarkers that could be used to identify certain pathogens. Hyperspectral remote sensing data can complement UV-VIS-NIR spectroscopy data. This could help to create algorithms that would analyze the influence of natural light on hyperspectral remote sensing data. Raman spectroscopy data can be combined with FTIR spectrometry data, which complement each other perfectly, although the latter cannot be widely used in plant protection due to technological limitations. We believe that data on the dynamics of metabolome changes obtained by GC-MS and LC-MS methods will further help to facilitate this task.

## 6. Conclusions

This review critically discussed the advantages and disadvantages of modern remote sensing technologies that are used for early plant disease diagnostics. It was shown how to overcome the insufficient accuracy in plant diseases diagnosis which comes from the lack of comparison of obtained remote sensing data and the biochemical processes occurring as a result of plant–pathogen interactions.

The great potential of Raman spectrometry and hyperspectral remote sensing for the non-destructive detection and diagnosis of plant diseases was shown. The advantages and disadvantages of both methods, as well as other spectroscopic and imaging techniques were revealed. It was shown that metabolomic approaches can help to identify groups of organic compounds in the studied diseased plant’s metabolome, to create biomarkers for early plant disease detection. Metabolomic analysis can be based on both chromatographic and spectral data, with Raman spectrometry being the most promising of the spectrometry techniques.

## Figures and Tables

**Table 1:** A comparison of the technical methods for plant disease monitoring and diagnosis.

Feature	qPCR	Hyperspectral Remote Sensing	Raman Spectrometry
Disease monitoring *	No	Undefined, probably yes	Undefined, probably yes
Disease diagnosis *	Yes	Undefined, probably yes	Undefined, probably yes
Consumables	Yes	No, but an aircraft is needed for large area monitoring	No
Sensitivity *	High	Undefined, probably medium or high	Undefined, probably high
Specificity *	High	Undefined, probably medium or high	Undefined, probably high
Early disease detection *	Yes, but depends on proper monitoring; other way is too expensive.	Undefined, probably yes	Undefined, probably yes
Cost **	Bio Rad CFX Opus RT PCR USD 17.000 ***	Cubert Ultris 20 camera USD 50.000	Rigaku Progeny ResQ USD 12.000
Staff requirements	High	Medium	Low
Time of analysis	Medium	Medium	Low
Data analysis requirements	Medium	Low ****	Low
Portability	No	Yes	Yes
Destructiveness	Yes	No	No
Main advantages	High sensitivity and specificity. Proven method.	Can monitor large areas, especially using a satellite. Great potential for high sensitivity and medium specificity.	Very fast, may be used both for monitoring and diagnosing at the same time. Great potential for high sensitivity and specificity.
Main disadvantages	Useless if there are no available probes for the specific infection. Needs preliminary monitoring of the disease. Requires qualified personnel and quite expensive consumables.	At this point, the possible need to use two different cameras to cover the entire spectral range. High price of sensors. Potentially low sensitivity and specificity, due to physical aspects of leaf–light interactions.	It is currently unknown whether the use of Raman spectroscopy will allow the detection of all specific metabolites that can be used to detect plant diseases, since some molecules are poorly detected by these sensors.
	A proven method for plant disease diagnosis that does not yet have alternatives, but has a number of disadvantages.	Best overall choice for disease monitoring, especially if based on a satellite platform.	In summary, may become the best method for early plant disease diagnosis: non-invasive, accurate, fast, and cheap.

* Must be confirmed by available commercial products; ** according to open data; *** the cost of consumables is taken into account separately; **** low if there are automatic data processing systems, but otherwise is very high.

**Table 2:** The compounds mentioned in articles on Raman spectrometry.

Compound	Metabolite Type	Total Mentions	References
Aldehydes	Primary or Special	2	[130,166]
Aliphatics	Primary or Special	50	[142,144,145,147,148,149,152,176,177,178,179]
Aromatics	Primary or Special	1	[143]
Carboxyllic acids	Primary or Special	4	[142,152,177]
Carbohydrates	Primary	67	[142,145,147,149,150,151,152,176,177]
Carotenoids	Primary	37	[142,143,144,145,147,148,149,150,151,152,176,177,178]
Chlorophylls	Primary	12	[143,149]
Celluloses	Primary	41	[142,144,145,147,148,149,150,152,176,177,178]
Esters	Primary or Special	2	[142,177]
Flavonoids	Special	2	[149]
Ketones	Primary or Special	2	[142,177]
Lignins	Primary	41	[142,143,144,147,148,149,150,151,152,176,178]
Luteins	Special	1	[143]
Pectines	Primary	9	[142,144,147,148,152,176,177,178]
Phenolics	Primary or Special	6	[143,149,178]
Phenylpropanoids	Primary or Special	15	[144,145,147,177]
Proteins	Primary	20	[142,145,147,148,149,150,151,152,176,177,178]
Terpens	Special	3	[149,152]
Xylans	Special	13	[142,144,147,148,152,176,177,178]

## Data Availability

Not applicable.
